# Relevance theory and the social realities of communication

**DOI:** 10.3389/fpsyg.2023.1167790

**Published:** 2024-01-29

**Authors:** Marilynn Johnson

**Affiliations:** Department of Philosophy, University of San Diego, San Diego, CA, United States

**Keywords:** Relevance Theory, testimonial injustice, communication, Grice, Sperber and Wilson, Fricker, credibility

## Abstract

A central tenet of theories of meaning in the Gricean tradition—such as Relevance Theory—is that others will come to believe certain things simply by recognizing our intentions to communicate. In this article I demonstrate that this is not equally the case for all interlocutors; some bear additional burdens. In particular, I argue that this can happen in two ways. First, I demonstrate how a response to persistent testimonial injustice can be understood in terms of Sperber and Wilson’s distinction between meaning-that and showing-that; a speaker who experiences repeated testimonial injustice will often move down the meaning vs. showing continuum. This is a result of a speaker learning that recognition of her intention has not in her experience been sufficient to induce the intended response in the hearer. Secondly, in consideration of social science research around perception of accent prestige and other status cues, I detail further costs borne by those who change their physical appearance and voice to be perceived as more credible. The costs of communication are not equal for all: they are greater for those who face a credibility deficit based in identity prejudice. Overall, by bringing Fricker’s notion of testimonial injustice to bear on Relevance Theory, this article shows how social factors affect the reality of how interlocutors communicate.

## Introduction

A central tenet of theories of meaning in the Gricean, pragmatic tradition—such as Relevance Theory—is that others will come to believe certain things by recognizing our intentions to communicate. I argue that those working in this tradition need to consider the additional burden that is borne by some interlocutors in getting others to come to believe some content. I will demonstrate how a response to persistent testimonial injustice can be best understood in terms of a distinction presented by Dan Sperber and Deirdre Wilson between meaning-that and showing-that. I argue that a speaker who experiences repeated Testimonial Injustice may respond by moving down Sperber and Wilson’s meaning vs. showing continuum. This explains an additional downstream effect not explicitly discussed by Miranda Fricker in her work on Testimonial Injustice. I then will present my understanding of what I call “social interpretation.” In consideration of social science research around perception of prestige and status cues, I detail further costs borne by those who undertake the rational process of making changes to their physical appearance and voice to be perceived as more credible. I will conclude with what I see as the main takeaways from my argument.

This paper presents a socially-situated account of philosophy of language. In this focus, I follow work by philosopher Miranda Fricker who writes, “a socially situated account of a human practice is an account such that the participants are conceived not in abstraction from relations of social power…but as operating as social types who stand in relations of power to one another” (Fricker, 2006, p. 3). This socially situated account stands in contrast to how much philosophy of language is usually conducted. It will come as no surprise to philosophers of language that the field has for the most part ignored these social realities of communication. But this might be news to those outside the debates within the discipline. For language is one of the ways that class, race, and power are most evident.

Ignoring this reality, examples in much philosophy of language literature are given in terms of “interlocutors” or discussions between people with names like “Smith” and “Jones,” “Mary” and “Paul.” What is the race of these interlocutors? What is their social status? What is their gender? Of course, in philosophy there is a certain amount of “compulsory rational idealization” that is necessary in presenting theoretical frameworks (Fricker, 2006, p. 2). However, in pragmatics—the branch of philosophy of language that seeks to turn away from abstract discussions of language itself and consider the reality of how we communicate with each other—we should aim to eventually turn away from abstraction and develop more fully-fleshed out accounts of the messy social realities that shape communication.

I will assume an intentionalist account of meaning as a starting place for this article. Of course, some reject an intentionalist account of meaning; but, defending intentionalism will not be my focus here (for such a defense see Johnson, 2019; Johnson, 2022a). Here my focus is a discussion of Relevance Theory, which falls within the Gricean, intentionalist tradition. I should also specify that my arguments here are not presented as a criticism of either the Sperber and Wilson or Fricker positions—but rather as a fruitful way of building on both of their theories by bringing them together.

## Meaning and showing

Let me now commence with presenting the relevant parts of Sperber and Wilson’s theory. Sperber and Wilson’s Relevance Theory was first presented in their 1986 *Relevance: Communication and Cognition* and they have continued to further develop their position since that time. One recent expansion on content from that book was their 2015 paper “Beyond Speaker’s Meaning” in which they defend Relevance Theory broadly and develop further some theoretical machinery. Sperber and Wilson argue that their theory best captures what we want from a theory of communication—i.e. is more “conceptually unified,” picks out “the proper object of a philosophical definition or a scientific theory,” and “makes good sense of our fuzzy intuitions about speaker meaning” (Sperber and Wilson, 2015, p. 117).

Relevance Theory can be seen as a part of the Gricean tradition in that it follows in the footsteps of philosopher of language H. P. Grice, whom Deirdre Wilson studied with at Oxford. Relevance was one of Grice’s proposed four maxims of conversation but the way relevance is understood by Sperber and Wilson is quite different. For them relevance is the key to ostensive-inferential communication. As they write, “By producing an utterance, the speaker requests the hearer’s attention. By requesting his attention, she suggests that her utterance is relevant enough to be worth his attention. This applies not just to speech but to all forms of ostensive communication” (Sperber and Wilson, 1986, p. 154). By ostensive communication they mean other nonverbal acts such as pointing to a clock, or ringing a doorbell (Sperber and Wilson, 1986, p. 53; Sperber and Wilson, 2015).

Although they are part of the Gricean, pragmatic tradition, Sperber and Wilson depart from Grice in a number of other important ways (see Sperber and Wilson, 1986, p. 161–163, Carston, 2005, and Horn, 2006 for further discussion of how Relevance Theory relates to Grice). In presenting his account of meaning, Grice argues for a definition with three conditions—including a third clause that recognition of the speaker’s intention be in some way *the basis* for a hearer to produce the intended response. In contrast, Sperber and Wilson prefer to work with a more “permissive” account that drops this requirement leaving only the first two. With the third clause dropped this picks out what they call “ostensive communication.” They write,

In characterising ostensive communication, we built on the first two clauses of Grice’s definition and dropped the third. This was not because we were willing to broaden the definition of utterer’s meaning—we agreed with Grice that talk of ‘meaning’ is awkward in certain cases—but because it seemed obvious that there is a continuum of cases between ‘meaning that’ (typically achieved by the use of language) and displaying evidence that (in other words, showing) and we wanted our account of communication to cover both (Sperber and Wilson, 2015, p. 119).

Whether it is better to work with Grice’s original three clauses or drop them in favor of two is something that is debated amongst those working in the pragmatic tradition.^1^

By dropping Grice’s third clause, Sperber and Wilson open up the sorts of relevant cases to include “meaning that” as well as “showing that,” which they then define. They write that meaning that (MT) is “typically achieved by the use of language” and that showing that (ST) is “displaying evidence that” (Sperber and Wilson, 2015, p. 119). By dropping Grice’s third clause the Sperber and Wilson account covers a wider range of communicative acts, including those cases where the intention to communicate is superseded by the direct evidence. For instance, when presented with direct evidence of some fact, such as that I have a bad leg, recognition of my intention is no longer a *reason* to come to believe some proposition, such as that I cannot play squash. For Sperber and Wilson this would be a case of ostensive-inferential communication; for Grice it would not be a case of non-natural meaning.

In other words, the Sperber and Wilson account can be understood as explaining the various ways to get others to believe certain things or behave in certain ways, including those where recognition of an intention is not necessary. Sometimes we do expect intention recognition (with MT utterances), and sometimes we display direct evidence (with ST), as captured by the Sperber and Wilson MT-ST continuum.^2^

As a last bit of relevant theory before moving on to some motivating examples, let me also note that Sperber and Wilson present their account in terms of manifestness, understood as a technical term. When some content p is shown or meant, this is the sort of thing that makes p more manifest on the Sperber and Wilson picture. Manifestness is a combination of epistemic strength and salience. Manifestness is the extent to which, for any given proposition, the interlocutor “is likely to some positive degree to entertain it and accept it as true” (Sperber and Wilson, 2015, p. 134). ‘Salience’ here is what they called ‘accessibility’ in their original 1986 presentation in *Relevance* (Sperber and Wilson, 2015, p. 133). Manifestness is a property of the proposition itself, given the context.^3^

## Some motivating examples

Before moving on to the Fricker and social psychology literature discussion, let me now give 3 anecdotes that illustrate the difference between meaning that (MT) and showing that (ST). The first and second examples have me as the speaker, i.e., the one wishing to persuade in the exchange. In the third example I am the hearer, i.e., the one being persuaded in the exchange. I will first present the examples and then explain their relation to the Sperber and Wilson MT-ST continuum.

### Motivating example Case 1

Last spring I ordered a bracelet online. The package came on time as expected. I opened the sealed shipping box. There was no indication it had been opened. The shipping box contained a velvety bag, which contained a small padded box. The small padded box was empty. Strangely, it contained the price tag that should have been attached to the bracelet. The box had apparently not been tampered with so it seemed like the issue originated when it was packed. I wanted my bracelet or a refund for the money. I called the relevant customer service number and described the situation to them. I knew it sounded strange—because it was in fact strange. The person I spoke with on the phone asked me to send them a picture of the empty box and I did. They accepted this as satisfactory and sent me a new bracelet.

### Motivating example Case 2

Last summer I ordered 6 dresses online, persuaded by some huge end-of-season markdowns. I had a big event coming up and thought perhaps one of them would be suitable. I happened to be outside for the delivery and I accepted the box directly from the FedEx delivery man. When I got inside I noticed that the box was very squished. The original brown tape that sealed the top was opened and it had been haphazardly taped again with clear tape. I opened the box to find 2 of the 6 dresses inside.

My thinking here was shaped by my previous experience with the bracelet where I had been asked to send a photo. I saved the box—now I had some evidence that could show it had been opened and then resealed. I called the customer service number and explained the situation. They said there would be an “investigation.” It did not sound promising. Next thing I knew I had a refund for the full purchase price to my credit card—so I ended up getting 2 dresses for free. I never needed the damaged box as evidence so I recycled it.

### Motivating example Case 3

A few years ago, I received an email from a student saying that she could not come to class because she had jury duty. Any professor is familiar with emails of this sort and we usually get multiple of them each week. My standard response, as I believe is the case for many other professors, is to let the student know I appreciate them reaching out and tell them that they should get the notes from a classmate and come to office hours if they have questions. If the student says they are sick I also tell them I hope they feel better soon. In almost all cases it does not matter to me if they are lying, and realistically I know a certain percentage will be. I emailed this standard response to the student who said she had jury duty. My student then replied again with a photo of her jury summons. I had not asked her for it.

## Discussion of cases and MT-ST continuum

In Case 1, I tried to get the customer service agent to believe that my bracelet had been missing from the package. I told her this verbally—a case of MT. This was not sufficient and she asked me to ST—provide “evidence”–and so I sent the photo of the empty box. In Case 2 I again tried to get the customer service agent to believe that 4 of my items had been missing from the package. I told her this verbally—a case of MT. This was sufficient and I was not asked to ST—to send a photo of the box.

However, in Case 2 I still incurred the cost of saving the box. I was less confident that I would be believed just on the basis of my word. My expectation had been shaped by my previous experience where I first tried to get the agent to believe something with my word alone. Since this wasn’t enough I prepared to show evidence in a similar interaction in the future.

In Case 3 the student first MT when she told me she had jury duty. The student ST when she sent the photo, providing me with direct evidence. I had not asked her for this photo. What exactly had caused her to make this shift? Did she send me the picture of her jury summons because she thought I believed she was lying? Just like me with the customer service agents, she likely had experienced a similar situation in the past. She likely had had a professor or teacher who did not accept her word as enough and asked for some sort of proof. She evidently thought that my response meant that I needed further documentation and thus provided it.^4^

As can be seen in Cases 1, 2, and 3, moving down the meaning-showing continuum can be a result of a speaker learning that recognition of her communicative intention has not in her experience been sufficient to induce the intended response in the hearer.

## Social interpretation

The costs of communication are not equal for all interlocutors—they may be greater for those who must show what they wish to make manifest to their hearers. Why would someone, on an occasion, choose to provide direct evidence in support of some fact rather than expect that their communicative intention alone would be enough to make some content manifest in the hearer? The answer has to do with how they expect they will be interpreted. If we reflect on social realities it becomes clear that manifestness as Sperber and Wilson define it—the extent to which, for any given proposition, an interlocutor “is likely to…accept it as true” (134) depends not just on the proposition itself but on *who says* that statement to us.

In presenting their account of relevance, Sperber and Wilson do hint at the role of power dynamics in their notion of optimal relevance to an individual^5^ (Sperber and Wilson, 1986, p. 142–161). They write, “How much effort the addressee can expect the communicator to put into being relevant varies with the circumstances, the communicator, and the relationship between communicator and addressee” (Sperber and Wilson, 1986, p. 160). Later in the paragraph they illustrate this point noting that “A master talking to his servant may say whatever he wishes and merely assume that it will be relevant enough” (160). They also illustrate the point with an example of a woman named Mary who is to infer that she should make dinner when her surgeon husband says “I had a long day. I’m tired” (145–149). As they describe, when we engage in communication of this sort with well known interlocutors, we can bring an array of background assumptions to bear on the conversation. These mentions of a power dynamic do not receive further treatment but Sperber and Wilson are explicit to note that characterization of relevance to an individual is “psychologically more appropriate” (142).

These background assumptions about our interlocutors develop over time into what we might call a more or less refined “theory” about the speaker. Sometimes these “theories” are based on extensive knowledge of past interactions and other times they rely on rough heuristics. In the canonical paper “A Nice Derangement of Epitaphs” philosopher David Davidson draws our attention to such socially-relevant features of a speaker. As he notes an interpreter “alters his theory” about a speaker in light of these factors:

An interpreter has, at any moment of a speech transaction, what I persist in calling a theory. (I call it a theory, as remarked before, only because a description of the interpreter’s competence requires a recursive account.) I assume that the interpreter’s theory has been adjusted to the evidence so far available to him: knowledge of the character, dress, role, sex, of the speaker, and whatever else has been gained by the speaker’s behavior, linguistic or otherwise. As the speaker speaks his piece the interpreter alters his theory (Davidson, 2006, p. 260).

Let us take it as a given that Davidson has made an important point about the social realities of communication—which are often overlooked by philosophers of language. Davidson does not specify exactly how an interpreter would alter his theory in light of each of these factors, but we can now turn to philosopher Miranda Fricker to consider some specific relevant examples of just this very thing.

In her work Fricker presents a “socially situated account,” which, again, she defines as “an account such that the participants are conceived not in abstraction from relations of social power…but as operating as social types who stand in relations of power to one another” (Fricker, 2006, p. 3). Fricker’s account of the aims of testimony bears striking similarities to the Sperber and Wilson notion of manifestness and what Davidson discusses in the section just quoted above. Fricker explains,

We are picturing hearers as confronted with the immediate task of gauging how likely it is that what a speaker has said is true. Barring a wealth of personal knowledge of the speaker as an individual, such a judgment of credibility must reflect some kind of social generalization about the epistemic trustworthiness—the competence and sincerity—of people of the speaker’s social type, so that it is inevitable (and desirable) that the hearer should spontaneously avail himself of the relevant generalizations in the shorthand form of (reliable) stereotypes (Fricker, 2006, p. 32).

Gauging how likely it is that what a speaker has said is true in “face-to-face testimonial exchanges” requires a the hearer to, as Fricker writes, “make some attribution of *credibility* regarding the speaker. Such attributions are surely governed by no precise science, but clearly there can be error in the direction of excess or deficit” (Fricker, 2006, p. 18). Manifestness in the Sperber and Wilson sense clearly is not just a matter of the content of some proposition—it also depends who asserts this content to us. And it should: we should not take all people to be equally reliable sources of information, indiscriminately changing our beliefs regardless of who is the source. As Fricker writes, “Much of everyday testimony requires the hearer to engage in a social categorization of speakers, and this is how stereotypes oil the wheels of testimonial exchange” (Fricker p. 32). When faced with interpretive knowledge gaps we need to fill them in somehow.

To illustrate her points Fricker has us consider a case of the dependable family doctor (Fricker, 2006, p. 32). Consider the following utterance said by a family doctor:

“You will be at increased risk of heart attack if you get the new COVID-19 booster.”

And consider again the utterance said by the person sitting next to you on the last airplane you took. We would likely give different weight to this utterance about COVID-19 boosters based on who said it.

Picture now very clearly that reliable family doctor. Get a fleshed-out mental picture. Consider now the gender, race, and accent of the family doctor you were picturing. What Fricker draws particular attention to in her work is the way that identity prejudice can be present in otherwise rational assessments of speaker credibility. She writes, “Many of the stereotypes of historically powerless groups such as women, black people, or working-class people variously involve an association with some attribute inversely related to competence or sincerity or both: over-emotionality, illogicality, inferior intelligence.” (32) We do not fill in those gaps in the same way for all speakers.

Fricker calls “Testimonial Injustice” when “prejudice causes a hearer to give a deflated level of credibility to a speaker’s word” (2007, p. 1). She vividly illustrates what Testimonial Injustice looks like with a discussion of Harper Lee’s *To Kill a Mockingbird*.

The year is 1935, and the scene a courtroom in Maycomb County, Alabama. The defendant is a young black man named Tom Robinson. He is charged with raping a white girl, Mayella Ewell, whose family’s run-down house he passes every day on his way to work, situated as it is on the outskirts of town in the borderlands that divide where whites and blacks live. It is obvious to any reader, and to any relatively unprejudiced person in the courtroom, that Tom Robinson is entirely innocent. For Atticus Finch, our politely spoken counsel for the defense, has proved beyond doubt that Robinson could not have beaten the Ewell girl so as to cause the sorts of cuts and bruises she sustained that day, since whoever gave her the beating led with his left fist, whereas Tom Robinson’s left arm is disabled, having been injured in a machinery accident when he was a boy. The trial proceedings enact what is in one sense a straightforward struggle between the power of evidence and the power of racial prejudice, with the all-white jury’s judgment ultimately succumbing to the latter (Fricker, 2006, p. 23).

Fricker presents this case as a “struggle between the power of evidence and the power of racial prejudice.” We also see illustrated in this case a struggle between MT and ST. In claiming that Tom Robinson raped her Mayella Ewell is able to MT and be believed. Tom Robinson, through his lawyer Atticus Finch, knows that to simply MT in reply will not lead the jury to believe that Tom is innocent. He must provide direct evidence.

Lawyers in presenting their cases do sometimes rely on MT. They coach witnesses on how to appear credible and bring in experts (Loftus and Ketcham, 1992; Elm, 2008). But in Tom’s case—given how he will be perceived as an African American man at this time in America—ST is needed. Atticus in representing his client moves down the MT-ST continuum. As readers who know his innocence we hope this will be enough. But it still is not. As Fricker writes, “They fail, as Atticus Finch feared, precisely in their duty to believe Tom Robinson” (26).

Of course, most situations in which we try to convince someone of some proposition are not played out in the court of law, but in more informal circumstances. We do see parallels however in “the court of the professor’s decision” and “the court of the customer service representative.” Depending on the stereotypes we have about a speaker they will sometimes be able to persuade with MT, sometimes with ramping things up to ST, and sometimes not even ST will be enough.

Fricker’s observations, as she notes, are borne out not just by fictions such as *To Kill a Mockingbird*, but by social psychology research as well. Fricker cites psychologist Taylor (1982) who writes, “Empirical work on non-social judgments indicates that the perceiver employs shortcuts or heuristics to free capacity and transmit information as quickly as possible.” Fricker notes that this need not be conscious or deliberate, citing Kahneman and Tversy (1973), whose work on System 1 and System 2 shows a number of the mental shortcuts that we make every day, and the ways they are subject to systematic and predictable errors (Kahneman, 2013). For instance, after being presented with an anchor of some number, participants when then asked to estimate some quantity are more likely to give a figure closer to that anchor than those who have not been primed in this way (Kahneman and Tversy, 1973; Kahneman, 2013).

Again, circling back to the quotation by Davidson, not all speakers are perceived in the same way. The speaker’s “the character, dress, role, sex, of the speaker, and whatever else has been gained by the speaker’s behavior, linguistic or otherwise” (Davidson, 2006) can serve as a sort of “anchor” for how much credibility they are afforded by a hearer.

Accent is one of the clearest ways that credibility can be affected in the eyes of the interpreter, and there has been much empirical research conducted on this topic (Dixon et al., 2002; Kinzler et al., 2007; Lev-Ari and Keysar, 2010; Dragojevic et al., 2021). There are a number of ways that accents can be classified and they can signal class, race, gender, country of origin and many other things. One way that researchers Dragojevic et al. classified accents in a recent summary paper on one hundred years of language research is as “low prestige” and as “high prestige” (Dragojevic et al., 2021). They write that, “Research shows that language varieties within a given society can be ordered on a hierarchy of prestige, typically corresponding to the socioeconomic status of the social groups they are associated with. Varieties associated with socioeconomically dominant groups tend to carry high prestige; these typically include majority group languages, standard varieties—namely those that have been codified” (Dragojevic et al., 2021, p. 63). They define low prestige varieties of language as those we “associate with socioeconomically subordinate groups,” and continue to note that “these typically include minority group language, nonstandard varieties—namely those that diverge from codified norms, including most regional and ethnic dialects and foreign accents—and other forms linked to stigmatized groups (e.g., gay/lesbian speech)” (Dragojevic et al., 2021, p. 63). As we might expect, their summary of one hundred years of language research shows that “Speakers of low prestige varieties [of language] frequently face prejudice and discrimination” (Dragojevic et al., 2021, p. 67). This is just the sort of thing Fricker draws our attention to in her work.

Further, this bias against low prestige varieties of language is found even earlier than one might expect. As Dragojevic et al. note, at 5 months infants can distinguish between native and foreign accents and “express a clear social preference for native- over foreign-language speakers, without any knowledge of specific linguistic stereotypes” (Kinzler et al., 2007; Dragojevic et al., 2021, p. 63). At age 10 to 12 months infants are “more likely to accept toys from native over foreign language speakers” and preschoolers trust native-language friends more than foreign-language friends (Kinzler et al., 2007).

One place where older children might learn this bias against non-native speakers is in the media, if they are not already exposed to it in their everyday life. For, in an analysis of Disney movies, cartoon, and primetime television it was found that “standard speakers tend to be portrayed in positive roles, whereas nonstandard speakers—particularly foreign-accented speakers—in negative roles” (Dragojevic et al., 2021, p. 67). From childhood we are conditioned to trust certain speakers less than others. Unsurprisingly, this carries over into adulthood, where nonstandard speakers “tend to be judged as less credible, truthful, and accurate eyewitnesses^6^” (Dragojevic et al., 2021, p. 68). Accent cues are just one of the many ways that an interlocutor can have a “credibility excess” or “credibility deficit.”

There are many other cues present in speech and bodies including but not limited to the perception of the speaker’s race, gender, class, and age. In addition, a speaker’s vocal pitch and pacing affect how they are perceived. Vocal pitch is associated with size in humans and animals (Sell et al., 2010). Empirical research has demonstrated that vocal pitch in both men and women is correlated with perception of leadership quality, attractiveness, and strength (Zuckerman and Miyake, 1993; Sell et al., 2010; Klofstad et al., 2012). In a study on pitch and politics the authors conclude that “because women, on average, have higher-pitched voices than men, voice pitch could be a factor that contributes to fewer women holding leadership roles” (Klofstad et al., 2012). Acoustic analysis has demonstrated that certain linguistic features are associated with trustworthiness, independent of attribution of a gender to the speaker, including “accelerated tempo, low harmonic-to-noise ratio, more shimmer, low fundamental frequency, more jitter, large intensity range” (Schirmer et al., 2020). Physical bodily features also affect how a “credibility excess” or “credibility deficit” is attributed to interlocutors. Those who are “babyfaced” are thought to be less competent (Zebrowitz and Montepare, 2005). Taller people are thought to be more natural leaders and earn more money (Judge and Cable, 2004; Maclean, 2019). The taller candidate has won the U.S. election two-thirds of the time (Maclean, 2019).

When speakers are perceived to have certain features of any kind that give them a credibility deficit, it is rational to do a number of things to lessen these appearances. These can include working to change one’s accent or style of dress.

These changes made to be perceived as a different sort of interlocutor are prevalent. We see this reflected in fictions such as Eliza Doolittle in the famous Shaw play *Pygmalion*, and with characters such as Lucien de Rubempré in Balzac’s *Lost Illusions.* These fictions ring true because they capture a reality that persists today.

Given the knowledge that how we appear changes how likely we are to be believed, it is only rational to make changes to be perceived more favorably. Being overweight is associated with being poor and uneducated and thus it is “economically rational for ambitious women to try as hard as possible to be thin” (Economist, 2022). Adorning the body in a way that changes the perception of the physical body and thus the associated meanings is what I have called in other work imitation of natural meaning (Johnson, 2022a), drawing here on Grice’s distinction between natural and non-natural meaning. This has been seen throughout history and can happen in ways large and small, from dying one’s hair, to wearing a suit, to wearing makeup (Johnson, 2022a). Female politicians are coached so as to appear feminine to the right degree, down to details like changing what they wear, being coached on the pitch of their voice, and how to reduce small gestures such as touching their hair, which are perceived negatively (Jahnke, 2011). Attorneys, too, pay great sums of money to jury consultants who coach them on how they are perceived—often leading to feedback that is deemed “superficial” by attorneys but which substantially affects jury rulings, such as that an attorney needs to smile more or less (Kressel and Kressel, 2004, p. 4; Postal, 2022).

This is not to say that these changes are without costs—financial as well as emotional—or that this is the way that things ought to be. Many of these efforts to reduce an appearance that lead to a credibility deficit will be quite taxing (Du Bois, 1903/2016; Jahnke, 2011; McCluney et al., 2019), and reflect the sexism, racism, ableism, and other of the worst biases in our society. However, as individuals it is often wise to act prudentially with how we present ourselves to the world [distinguishing this from the times where we have a moral obligation to resist these stereotypes (Jeffers, 2012; Cray, 2021; Johnson, 2022b)]. On top of this, such efforts are unlikely to be entirely effective—presenting one’s self in certain ways can lessen the effect of the credibility deficit but it will usually not be fully eradicated. It also can lead to other costs: charges of being a traitor to one’s community (Barnes, 2022).

Speakers who are perceived to have a credibility deficit may move down the MT-ST continuum, as we have seen. I found myself doing this with what I learned between Case 1 and 2, and speculated that it is what motivated my student in Case 3. However, this is not the only change it would be rational for them to make. Recall that manifestness is an explicitly epistemic notion, the extent to which, for any given proposition, the interlocutor “is likely to some positive degree to entertain it and accept it as true” (Sperber and Wilson, 2015, p. 134). Through the discussion I have presented here we see two specific types of response to Testimonial Injustice emerge—the first understood as a move down the MT-ST continuum and the second understood as the steps taken to be perceived as more credible in the first place, to increase the likelihood that one’s MT will be enough. These are both a result of a speaker learning that recognition of her intention has not in her experience been sufficient to induce an intended response in the hearer.

We see the following two categories emerge:

1. **“Prove it” or Showing-That Injustice** – This is when a communicator expends extra time and resources presenting an interlocutor with direct evidence for some proposition that—barring identity prejudice—they would accept without such direct evidence.
2. **“Look it” or Personal-Appearance-Modification Injustice** – This is when a communicator expends extra time and resources presenting themselves in a way that makes them be perceived in a way that lessens their credibility deficit. This includes all forms of changes in adornment and bodily styling, as well as changes to accent, vocabulary, and manner of speech.

On the part of the speaker^7^ Showing-That and Personal-Appearance-Modification Injustice are rational response to past Testimonial Injustice, and includes both the intentional as well as automatic, unconscious changes.

## Takeaways

Many explanations of why we engage in communicative acts attempt to account for the cost of communication—an assumption that underpins Sperber and Wilson’s presumption of relevance.^8^ The potential for Fricker’s epistemic injustice theory to be applied directly to philosophy of language is made evident by Sperber and Wilson’s framework, as well as by their clear spelling out of manifestness as an explicitly *epistemic* notion. One of the types of moves we automatically take in processing information is to use stereotypes and heuristics of the sort that Fricker draws attention to in her work. This leads to discrepancies in the effort that different types of speakers have to expend in making their meanings manifest to interpreters. This leads to further forms of injustice because when certain speakers systematically face a credibility deficit they must expend more resources to be believed. This extends to the actions taken before making an utterance, as well as those that follow the recognition that a hearer requires direct evidence.^9^

In thinking about philosophy of language, we should be asking not only *how* but *why* we engage in certain forms of communicative behavior—such as why do we sometimes show and other times mean content. Often the answer to these types of questions lies in the details of social factors, of the sort that philosophers often gloss over in developing their theories. We should not ignore how questions of meaning and interpretation are shaped by the power dynamics at play between interlocutors. There is a time for abstraction; and, there is a time for addressing these social realities of communication.

## Author’s note

This paper has benefited enormously from the feedback of others. Thank you to the audience at the Philosophy of Linguistics and Language 2022 conference in Dubrovnik, especially Josh Armstrong, Michael Glanzberg, Stephen Neale, Peter Pagin, David Sosa, and Una Stojnic, as well as the reviewers for this journal for their detailed comments and suggestions. This paper would not have been written without the kind invitation for this Research Topic so I would like to thank the editors for their organizing efforts and support of the paper.

## Author contributions

The author confirms being the sole contributor of this work and has approved it for publication.

## Footnotes

^1^For instance, Stephen Neale discusses dropping the third clause in his often-cited 1992 paper on Grice (Neale 1992). Most scholars acknowledge that whether or not this clause is needed can be a matter of what one is aiming to capture with their theory. As Sperber and Wilson note in the quotation on this page the third clause is likely needed for certain cases of meaning. For more on this see Johnson (2019).

^2^In their 2015 paper Sperber and Wilson distinguish not only between meaning and showing but also between determinate and indeterminate content. For my purposes, I will be focusing on just determinate content, because otherwise the details of the theory become unwieldy. For further discussion of the Sperber and Wilson continua in all its complexity see Johnson (2019).

^3^Other related work including (Sperber et al., 2010; Sperber and Mercier, 2012, 2017, 2018; Mercier, 2020) discusses epistemic vigilance, trust, and reason. For our purposes I will not be adopting this epistemic vigilance framework and instead adopt the manifestness notion from the Sperber and Wilson (2015). There are a number of reasons for this. Most importantly, because of the fact that Sperber and Wilson explicitly use the notion of manifestness in their 2015 paper it is clear how they see it working with the rest of their framework on showing vs. meaning. Beyond this, epistemic vigilance is presented as a state of the interpreter rather than of the proposition in a context. Sperber et al. (2010) and Sperber and Mercier (2017, 2018) claim that epistemic vigilance is an evolved mental module. They state that epistemic vigilance is “typically conscious” and “involves engaging in some higher order or metarepresentational thinking about one’s own beliefs” (Sperber et al., 2010, p. 376). However, this sort of explicit reasoning on the part of an interpreter seems exceedingly rare. Seemingly aware of these issues, in their initial presentation of the notion of epistemic vigilance in 2010 the authors note that their paper included a number of assumptions “several of which we ourselves view as speculative” (Sperber et al., 2010, p. 384). Later formulations of this line of research (Sperber and Mercier, 2017, 2018) have not provided much needed clarity. Critics include Kim Sterelny, who noted that “appeal to a metarepresentational reasoning module seems not to help us at all” (Sterelny, 2018). Given such complications, I have chosen not to use the epistemic vigilance framework here. Again, because the notion of manifestness is a property of propositions in a context this requires positing no specific mental framework on the part of the interpreter.

^4^Case 3 is unlike Cases 1 and 2 in that I of course do not have direct access to the minds of my students and thus am forced to speculate here. I will say that as someone on the receiving end of her communications I did stop and think about what I was doing that caused her to communicate in this way. My thought process can be explained in terms of the Principle of Relevance. Here it seems that the student surely wanted to communicate something further than what she had already achieved with the first email, given that it is “mutually manifest that the communicator intends it to be manifest to the addressee that she has chosen the most relevant stimulus capable of fulfilling her intentions” (Sperber and Wilson, 1986, p. 157) and that “to the best of the communicator’s knowledge, the ostensive stimulus is relevant enough to be worth the audience’s attention” (Sperber and Wilson, 1986, p. 156).

^5^Although if that is what they had in mind with this case it is not made explicit. The terms ‘race,’ ‘gender,’ ‘power,’ ‘class’ are not found in the Appendix of Sperber and Wilson (1986).

^6^They write, “In simulated criminal proceedings, nonstandard speakers are often judged as more guilty than standard speakers…and as more likely to be re-accused of a crime, regardless of the quality of the evidence presented against them” (Dragojevic et al., 2021, p. 68).

^7^Although I focus on speakers here, in future work I hope to explore how these forms of injustice are also prevalent in those who want to be perceived as more credible hearers.

^8^They write, “The key problem for efficient short-term information processing is to achieve an optimal allocation of central processing resources. Resources have to be allocated to the processing of information which is likely to bring about the greatest contribution to the mind’s general cognitive goals at the smallest processing cost…Our claim is that all human beings automatically aim at the most efficient information processing possible” (Sperber and Wilson, 1986, p. 48–49).

^9^Relevance theory is a hearer-focused account but of course hearers are not operating in isolation from the speaker. Sperber and Wilson discuss the ways the relationship between hearers and speakers shapes relevance in context and consider the ways that speakers aim their utterances, and sometimes fail. e.g., see their discussion of bores on page 158 and the section on masters and servants gauging relevance in a context quoted earlier.
